# Biocontrol Potential of *Streptomyces hydrogenans* Strain DH16 toward *Alternaria brassicicola* to Control Damping Off and Black Leaf Spot of *Raphanus sativus*

**DOI:** 10.3389/fpls.2016.01869

**Published:** 2016-12-16

**Authors:** Rajesh K. Manhas, Talwinder Kaur

**Affiliations:** Department of Microbiology, Guru Nanak Dev UniversityAmritsar, India

**Keywords:** *Streptomyces hydrogenans* DH16, *Alternaria brassicicola*, *Raphanus sativus*, biocontrol, culture supernatant

## Abstract

Biocontrol agents and their bioactive metabolites provide one of the best alternatives to decrease the use of chemical pesticides. In light of this, the present investigation reports the biocontrol potential of *Streptomyces hydrogenans* DH16 and its metabolites towards *Alternaria brassicicola*, causal agent of black leaf spot and damping off of seedlings of crucifers. *In vitro* antibiosis of strain against pathogen revealed complete suppression of mycelial growth of pathogen, grown in potato dextrose broth supplemented with culture supernatant (20% v/v) of *S. hydrogenans* DH16. Microscopic examination of the fungal growth showed severe morphological abnormalities in the mycelium caused by antifungal metabolites. *In vivo* studies showed the efficacy of streptomycete cells and culture supernatant as seed dressings to control damping off of *Raphanus sativus* seedlings. Treatment of pathogen infested seeds with culture supernatant (10%) and streptomycete cells significantly improved seed germination (75–80%) and vigor index (1167–1538). Furthermore, potential of cells and culture supernatant as foliar treatment to control black leaf spot was also evaluated. Clearly visible symptoms of disease were observed in the control plants with 66.81% disease incidence and retarded growth of root system. However, disease incidence reduced to 6.78 and 1.47% in plants treated with antagonist and its metabolites, respectively. Additionally, treatment of seeds and plants with streptomycete stimulated various growth traits of plants over uninoculated control plants in the absence of pathogen challenge. These results indicate that *S. hydrogenans* and its culture metabolites can be developed as biofungicides as seed dressings to control seed borne pathogens, and as sprays to control black leaf spot of crucifers.

## Introduction

*Alternaria brassicicola* is one of the economically important pathogens worldwide with broad host range, and is established and widespread in many countries, including India (Verma and Saharan, 1994; Reis and Boiteux, 2010). It causes black spot disease and damping off of seedlings in *Brassica* spp. throughout the world and causes huge economic losses (Rimmer and Buchwaldt, 1995). Host plants can be affected at all developmental stages. Typical disease signs consist of black lesions on seedlings, leaves, stems, and siliquae resulting in accelerated senescence, premature pod shatter, and shrunken seeds (Mac Kinon et al., 1999; Iacomi-Vasilescu et al., 2004). Seeds infected with the pathogen result in reduced seed germination, photosynthetic potential, seedling vigor and pre- and post-emergence damping-off of seedlings (Nowicki et al., 2012), causing significant reduction in yield quantity and quality.

As a disease management strategy, the most feasible and economical option is the development of resistant brassicaceae crops. Unfortunately, transgenic approach used to develop resistant cultivars has proven to be a failure because of lack of expression of resistance in various varieties of crucifers (Sigareva and Earle, 1997). In many countries protection of crucifer crops from *A. brassicicola* is achieved using several different families of fungicides including dicarboximides, carbamates, benzimidazoles, and triazoles as seed or foliar treatments. However, in modern agriculture use of fungicides has become unpopular because of several problems related to environmental pollution, toxicity to humans, emergence of resistant strains and detrimental effects on non-target populations (Fox et al., 2007). So, there is a demand for new methods to supplement the existing disease management strategies to accomplish better disease control.

Biocontrol, as a part of integrated pest management, is a feasible replacement to the use of synthetic chemicals for more sustainable agriculture. The adaptiveness of most biocontrol agents to the environment in which they are used, the complexity of the organismal interactions and the involvement of numerous mechanisms of disease suppression by a single microorganism contribute to the belief that biocontrol will be more durable than synthetic chemicals (Sharma and Sharma, 2008).

Among microorganisms, actinobacteria, especially *Streptomyces* spp. are of utmost value as they are potent producers of bioactive compounds with different biological properties (Prabavathy et al., 2006) and have been established as potential biocontrol agents (Gomes et al., 2000; Ouhdouch et al., 2001). As reviewed by Berdy (2005) nearly 70–75% of secondary bioactive metabolites are isolated from these filamentous bacteria and 60% of the antibiotics that have been developed for agricultural industry are obtained from *Streptomyces* spp. (Tanaka and Omura, 1993).

Keeping in mind the importance of *Streptomyces* spp. as potent biocontrol agents, the present study was focused to evaluate *in vitro* and *in vivo* potential of *Streptomyces hydrogenans* strain DH16 (a strong antagonist toward various fungal phytopathogens, Kaur and Manhas, 2014) to control black leaf spot disease and damping off of *Raphanus sativus*, a major root vegetable crop worldwide with many health benefits.

## Materials and Methods

### Microorganisms

*Streptomyces hydrogenans* DH16 (GenBank: JX123130), a soil isolate having broad spectrum antifungal activity against fungal phytopathogens, was grown on starch casein nitrate agar (SCNA) slants and maintained at refrigeration temperature (4°C) for laboratory work. Mycelial fragments and spores of streptomycete were preserved in 20% v/v glycerol at -20°C as stock for future work. *A. brassicicola* MTCC 2102 was preserved on potato dextrose agar (PDA) slopes at 4°C.

### Production of Bioactive Metabolites by *S. hydrogenans* Strain DH16

For production of antifungal metabolites *Streptomyces* DH16 was grown on SCNA at 28°C. After 7 days of incubation, growth was scrapped and transferred into the starch casein nitrate broth to develop seed culture. After incubation (48 h), the production medium (starch: 12 g/l; soybean meal: 2.5 g/l; K_2_HPO_4_: 1.8 g/l; casein: 0.3 g/l; MgSO_4_: 0.1 g/l; FeSO_4_: 0.01 g/l; NaCl: 2.0 g/l; CaCO_3_: 0.02 g/l) was inoculated with seed culture and fermentation was carried out by incubating at 28°C at 180 rpm. After 3 days of incubation, filter sterile cell free supernatant was used for further experiments.

### *In vitro* Antagonistic Activity

*In vitro* antagonistic activity of 3 days old cell free supernatant was checked against *A. brassicicola* by well diffusion method (Kaur and Manhas, 2014).

### Suppression of Mycelial Growth of *A. brassicicola* in Broth

The effect of cell free supernatant of *S. hydrogenans* strain DH16 on mycelial growth of *A. brassicicola* was further determined in liquid culture according to Li et al. (2011). The 3 days old culture supernatant at concentrations of 0, 0.5, 1, 5, 7.5, 10, and 20% was supplemented into potato dextrose broth (PDB, 50 ml). Each flask was inoculated with single mycelial disk of 6 mm diameter of test fungus and uninoculated SCN broth served as control. After 7 days of incubation at 28°C, mycelial dry weight was determined, and growth inhibition was calculated as follow:

$$\begin{array}{c} \frac{\left[(\mathrm{Weight}\;\mathrm{of}\;\mathrm{untreated}\;mycelium-Weight\;\mathrm{of}\;\mathrm{treated}\;\mathrm{mycelium})\right]}{\mathrm{Weight}\;\mathrm{of}\;\mathrm{untreated}\;\mathrm{mycelium}}\times \;100 \end{array}$$

The experiment was repeated twice with three replicas.

### Effect of Culture Supernatant on Fungal Morphology of *A. brassicicola*

The effect of cell free culture supernatant of streptomycete on morphology of *A. brassicicola* was studied microscopically. Mycelium of *A. brassicicola* was taken from periphery of the inhibition zone around the well (containing culture supernatant of streptomycete) and from control plate and placed on glass slide in a drop of sterile water. The coverslip was placed on the film and then visualized under bright field microscope at 40× (Olympus). Microphotographs were taken using a digital camera.

### Effect of *Streptomyces* DH16 Culture Supernatant on Spore Germination

To study the effect of culture filtrate on spore germination of *A. brassicicola*, 100 μl of fungal spore suspension made in PDB (10^5^ spores ml^-1^) were mixed with 100 μl culture filtrate of different concentrations (0, 0.5, 1, 5, 7.5, 10, and 20% v/v; prepared using uninoculated production medium) and incubated at 28°C. In control, culture filtrate was replaced with 100 μl of SCN broth. After incubation of 8 h, 50 μl of each suspension were placed on sterile glass slide. After placing coverslip, slide was observed under microscope by counting about 50 spores.

### Extracellular Conductivity

The effect of antifungal metabolites on cellular leakage was studied by determining the extracellular conductivity of supernatants obtained from mycelial suspensions (*A. brassicicola*) treated with culture supernatant of DH16 (Lee et al., 1998). To obtain mycelial growth of fungus. Erlenmeyer flask (250 ml) containing 50 ml of PDB was inoculated with single mycelial disk of 6 mm diameter from 5-day-old PDA plate of test fungus. After 3 days of incubation at 28°C in PDB, mycelial growth was collected and washed thoroughly with sterile double distilled water. Washed mycelium (3 mg) was then added to flask containing 20 ml of culture supernatant of DH16. Mycelial suspensions were centrifuged at 10,000 rpm for 10 min to obtain supernatants, first immediately after the addition of mycelium, second after 12 h and third after 24 h of treatment. The experiment was repeated three times. Electrical conductivity was measured using a conductivity meter.

### Direct Observations of Antagonistic Effects of *Streptomyces* DH16 on Spore Germination and Growth of *A. brassicicola* in Soil Environment

A buried slide technique was used to determine the effect of streptomycete on fungal pathogen, directly in soil environment (Stevenson, 1956). 50 g of soil was sieved, air dried and sterilized in 100 ml glass beaker by autoclaving for 30 min at 121°C. A 7 ml spore suspension (1 × 10^8^ cells) of streptomycete was prepared. Spores were then thoroughly washed with sterile distilled water and inoculated into the sterile soil and incubation of soil was done at 28°C for 7 days. A 10 ml spore suspension of 5 days old *A. brassicicola* (1 × 10^5^ spores/ml) was mixed with 100 ml of sterile molten agar (1.8%), and 1 ml of it was coated on sterile glass slides. After solidification of the agar layer, slides were carefully inserted vertically into the beakers containing sterile soil alone (control) or DH16 inoculated soil and incubated at 28°C. At the end of every stated period, i.e., 2, 3, 4, and 6 days, slides were removed and examined immediately under a compound microscope at magnification of 400×. The experiment was conducted twice.

### Efficacy of Culture Supernatant and Cells of *Streptomyces* DH16 As Seed Treatment against *A. brassicicola* to Control Damping Off

#### Seed Treatment

The biocontrol potential of cell free culture filtrate and cells of *S. hydrogenans* strain DH16 as seed treatment against *A. brassicicola* using radish seeds was determined. Seeds were surface sterilized by immersing in sodium hypochlorite (1%) for 10 min. and then washed repeatedly with sterilized distilled water. The sterilized seeds were first artificially infected with the pathogen prior to antagonist treatment. The seeds were immersed for 4 h in fungal spore suspension in presence of 1% carboxymethyl cellulose (CMC; 10^5^–10^7^ spores/ml). These pathogen infested seeds were further given second treatment *viz*. (i) soaked in different concentrations (5, 10, and 20% v/v) of culture supernatant of antagonist/(ii) soaked in cell suspension of antagonist prepared in 1% CMC (10^7^–10^8^/ml).

In another treatment, uninoculated sterilized seeds were treated with (i) 1% CMC only (control), (ii) cells of antagonist only (10^7^–10^8^), and (iii) cell free culture supernatant only. After 1 h of second treatment, all seeds were dried in laminar flow on a sterile filter paper and used for further experiments.

#### Blotter Test

The moistened blotters were first used to determine the effect of antagonist to reduce damping off on radish plants grown from artificially infected seeds. Thirty seeds per treatment were placed in Petri dishes (10 seeds per plate) already lined with moist filter paper and covered loosely with another filter paper. Number of germinated seeds, and healthy and diseased seedlings were recorded after incubation of 7 days at 28°C in the dark. Seedling vigor (V) was determined by measuring root and shoot lengths and was calculated according to the equation:

$$\begin{array}{c} V\;=\;(L_{s}\;+\;\mathrm{Lr})\;\times \;G \end{array}$$

Where L_s_ is average shoot length in mm and Lr is average root length in mm and G is % germination (Andresen et al., 2015). The experiment was repeated twice.

#### *In vivo* Pot Experiment

Same treatments were given to seeds as described above, except in case of culture supernatant. For pot experiments, culture supernatant at a concentration of 10% was used as it showed significantly better results in blotter test. Seeds were sown in pots containing autoclaved soil with 10 seeds per pot. The pots were kept under natural conditions (month of February, 20 ± 2°C, 14 h light/10 h dark) and were watered daily. Seed germination was recorded after 15 days of sowing on the basis of above ground hypocotyls. Emergence of healthy seedlings, and mean fresh and dry weights of emerged plants were recorded. Disease incidence on radish plants was determined after 28 days on the basis of percentage of diseased seedlings.

The ability of the strain DH16 for root and rhizosphere colonization (seeds treated with *Streptomyces* cells) was also determined. Roots (from 10 plants) were recovered from the pots 28 days after planting and were cut into 1 cm pieces and suspended in 1 ml of autoclaved distilled water. Similarly, rhizosphere soil (10 soil samples) adhered to roots was carefully removed, weighed (1 g) and serially diluted. Then 100 μl from root aliquots and soil dilution (10^-4^) were spread on SCNA plates and cfu/ml were calculated after incubation for 7 days at 28°C.

### Biocontrol Potential of Culture Supernatant and *S. hydrogenans* As Foliar Treatment against *A. brassicicola* to Control Black Leaf Spot

The biocontrol potential of cells of *S. hydrogenans* and its cell free culture supernatant against *A. brassicicola* was also studied as foliar treatments using whole plants. Surface sterilized seeds of radish were sown in pots containing autoclaved soil. After 15 days, the leaves were given different treatments (i) inoculation with 10 μl of fungal pathogen (10^5^ spores/ml; inoculated control), (ii) co-inoculation of pathogen (10 μl) and culture supernatant of antagonist (10 μl of 10%), (iii) co-inoculation of pathogen and cells of antagonist (10^7^–10^8^ cells/ml), and (iv) non-inoculated healthy control (water only).

In another treatment, cell suspension/culture supernatant of *S. hydrogenans* was also applied to soil containing uninoculated radish plants.

After 20 days of various treatments, the plants were uprooted, fresh and dry weights of plants were recorded. Disease incidence was determined on the basis of dry weight of plants compared to control plants. The experiment was repeated twice.

### Statistical Analysis

All the experiments were repeated twice and the data (expressed as the mean ± SD) obtained from these experiments were subjected to statistical analysis. Tukey’s *post hoc* test was done with the help of ASSISTAT (7.7 β) to compare the means.

## Results

### Suppression of *A. brassicicola* in Liquid Broth by Culture Supernatant

Present study demonstrated inhibition of mycelial growth of *A. brassicicola* grown in broth supplemented with culture supernatant of DH16. In comparison to control, the mycelial dry weight of pathogen in PDB was significantly lowered in the presence of culture supernatant and this suppression of mycelial growth was found to be depended on the concentration of bioactive metabolites present in the culture supernatant. More than 50% inhibition was achieved at concentration of 5% and complete inhibition of mycelial growth occurred at 20% antifungal metabolites (*P* = 0.05; **Table 1**). Furthermore, the change in pH of the spent medium varied between 6.5 and 7.15, which suggested that the resulted mycelial inhibition was not due to pH change.

**Table 1:** Effect of filter sterile culture supernatant of *Streptomyces hydrogenans* DH16 on mycelial growth of *Alternaria brassicicola* when supplemented in potato dextrose broth at different concentrations.

Concentration (%, v/v)	*A. brassicicola*		
	Dry weight ofmycelium (mg/ml)^a^	Growth inhibition (%)	Final pH^b^
Control	5.2 ± 0.19a	0.00a	6.7 ± 0.01
0.5	4.8 ± 0.82b	8.9 ± 0.05b	6.7 ± 0.02
1	4.1 ± 0.07b	22.0 ± 0.01c	6.5 ± 0.07
5	1.8 ± 0.37c	65.5 ± 0.12d	6.7 ± 0.04
7.5	1.1 ± 0.04c	78.5 ± 0.03e	6.8 ± 0.04
10	0.53 ± 0.02d	89.7 ± 0.12f	6.9 ± 0.007
20	0.00e	100.0 ± 0.00g	7.0 ± 0.05

*^a^Means ± SD of two independent experiments, followed by the different letters within a column are significantly different according to Tukeys Test with *P* ≤ 0.05. ^b^Final pH of the spent medium.*

### Effect of Culture Supernatant on Fungal Morphology

**FIGURE 1:**
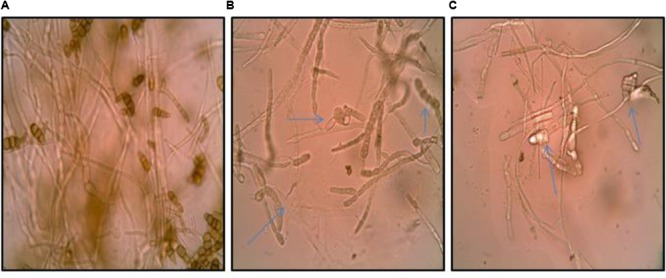
**Effect of culture supernatant of *Streptomyces hydrogenans* strain DH16 on *Alternaria brassicicola* mycelial morphology. (A)** Firm control mycelium **(B,C)** treated hyphae. Arrows indicate abnormalities like distorted mycelial structure, swellings, leakage of cellular material, loss of pigmentation in mycelium.

The inhibition of fungal pathogen by bioactive metabolites in the culture supernatant of the *Streptomyces* DH16 prompted us to examine the effect of its metabolites on the spore and mycelial structures. Microscopic studies demonstrated severe morphological abnormalities such as hyphal swellings resulting in bulbous structures, granular cytoplasm, leakage of cellular materials, thinning of hyphae, discoloration of hyphae, caused by metabolites present in the culture supernatant. Extracellular metabolites completely inhibited the sporulation along with loss of pigmentation (**Figure 1**).

### Effect of Culture Supernatant on Spore Germination

The effect of culture supernatant on spore germination was studied by incubating spores of *A. brassicicola* with different concentrations of supernatant. In control, 65% of the spores germinated after 8 h. At lower concentrations of 0.5 and 1%, the germination was not greatly affected as compared to control. However, it was significantly reduced to 25 and 10% at concentrations of 5 and 7.5%, respectively (*p* ≤ 0.0001). Concentrations of 10 and 20% were found to be completely lethal (no spore germination), and resulted in loss of pigmentation and shrinkage of spores.

### Extracellular Conductivity

**FIGURE 2:**
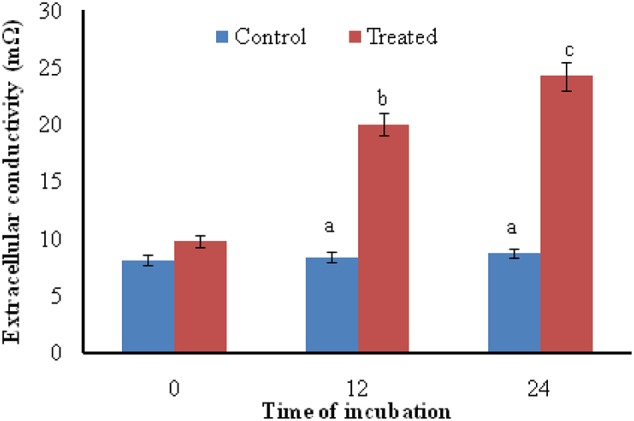
**Effect of antifungal metabolites on extracellular conductivity in suspension of test fungi (*A. brassicicola*) after 0, 12, and 24 h of treatment; Columns and bars represent the mean ± SD.** Same letters on the bar are not significantly different according to Tukey’s test (p ≤ 0.01).

Exposure of *A. brassicicola* to the antifungal metabolites of *S. hydrogenans* strain DH16 for 12 and 24 h resulted in increased levels of extracellular conductivity as compared to control, which showed leakage of cellular electrolytes from test fungus due to loss of cell wall/cell membrane integrity (**Figure 2**).

### Buried Slide Technique

**FIGURE 3:**
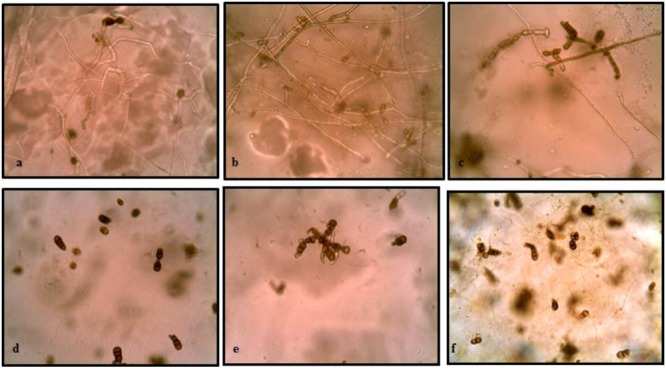
**Effect of soil inoculation with *S. hydrogenans* strain DH16 on germination of *A. brassicicola* spores.** Spore germination after 2 days in **(a)** uninoculated control soil **(d)** streptomycete inoculated soil (inhibited germination of spores); **(b)** formation of hyphal threads in control soil **(e)** small restricted germ tubes in inoculated soil, after 4 days; **(c)** New sporulation in control **(f)** distortion of germ tubes in treated soil, with further incubation upto 6 days.

The results of buried slide technique demonstrated 100% spore germination in control soil after 2 days of incubation (**Figure 3**). However, germination was significantly inhibited (only 7.14%) in streptomycete inoculated soil. With further incubation, germ tubes developed to form long hyphal threads in control soil where as in treated soil, small germ tubes of half the length of spores were formed in germinated spores. After 6 days incubation, complete mycelial structure with new sporulation was seen in control soil whereas in treated soil, germ tubes with loss of pigmentation and high vacuolization were observed.

### Biocontrol Efficiency of *Streptomyces* DH16 Cells and Culture Supernatant As Seed Treatment

#### *In vitro* Plate Assay

*In vitro* biocontrol potential of cells and culture supernatant of streptomycete against *A. brassicicola* was studied as seed treatment in radish. Seed germination, number of healthy seedlings and seedling vigor were found to differ significantly in treated and non-treated seeds (*P* ≤ 0.05; **Table 2**). In the seeds treated with pathogen alone, the percentage of seed germination and healthy seedlings, fresh and dry weights, and seedling vigor were significantly lower as compared to the uninoculated control seeds. On the other hand, treatment of pathogen infested seeds with culture supernatant at the highest concentration of 20% significantly improved seed germination and seedling vigor to 80% and 1538, respectively and were comparable to control. The percentage of healthy seedlings (90%) and their fresh and dry weights were also significantly higher in treated seeds. Similarly, treatment of pathogen infected seeds with antagonist also significantly improved all the parameters as compared to pathogen infested seeds. Additionally, seeds treated with streptomycete/culture supernatant only were found to be healthier than the uninoculated control seeds. Strain DH16 showed significant stimulatory effect and an increase of 21–35% over control in various growth parameters of seedlings was observed.

**Table 2:** Effect of *S. hydrogenans* strain DH16 and its metabolites on seed germination, growth of seedlings and seedling vigor in *R. sativus* during *in vitro* blotter assays carried out for 7 days

Seed treatment		Seed germination (%)	Healthy seedlings (%)	Fresh weight of seedlings (g)	Dry weight of seedlings (g)	Root length (cm)	Shoot length (cm)	Vigor index
Pathogen only		30 @ 1.1a	10.8 @ 0.66a	0.024 @ 0.08a	0.002 @ 0.04a	2.54 @ 0.5a	3.2 @ 0.40a	172.2 @ 1.5a
Pathogen and culture supernatant of different concentrations (%)	5	45 @ 2.5b	55 @ 1.0b	0.22 @ 0.01b	0.02 @ 1.0b	7.1 @ 0.76b	4.5 @ 0.36ab	517.5 @ 1.0b
	10	69 @ 1.13c	69.33 @ 1.13c	0.29 @ 0.11c	0.025 @ 0.08b	8 @ 1.75b	6 @ 1.15bc	966 @ 1.7c
	20	85 @ 1.5d	90.4 @ 2.0d	0.33 @ 0.15d	0.03 @ 0.5bc	9.4 @ 0.7bc	8.7 @ 0.5cd	1538 @ 2.0d
Pathogen and *S. hydrogenans* strain DH16 cells		75 @ 1.2e	74.0 @ 1.13e	0.30 @ 0.2c	0.028 @ 0.1b	8.46 @ 1.0b	7.1 @ 0.55bc	1167 @ 0.5e
*S. hydrogenans* strain DH16 cells only		88 @ 0.98f	100 @ 0.0f	0.40 @ 0.08e	0.042 @ 0.09c	12.02 @ 0.5c	10.28 @ 0.51d	1962 @ 0.98f
Culture supernatant only		90.0 @ 1.5g	100.0 @ 0.0f	0.38 @ 0.1e	0.033 @ 0.09c	11.5 @ 0.2c	10.0 @ 0.5d	1935 @ 0.1f
Untreated radish seeds		85 @ 1.02d	100 @ 0.0f	0.33 @ 0.06d	0.031 @ 0.12bc	9.72 @ 0.52bc	8.46 @ 1.1cd	1545 @ 1.02d

*Data represents Means ± SD of two separate tests (total 60 seeds for each treatment) where seeds were artificially infested with pathogen and then given different treatments with streptomycete cells and culture filtrate, different letters with in the column are significantly different according to Tukeys test; *p* ≤ 0.01.*

#### *In vivo* Pot Assay

**FIGURE 4:**
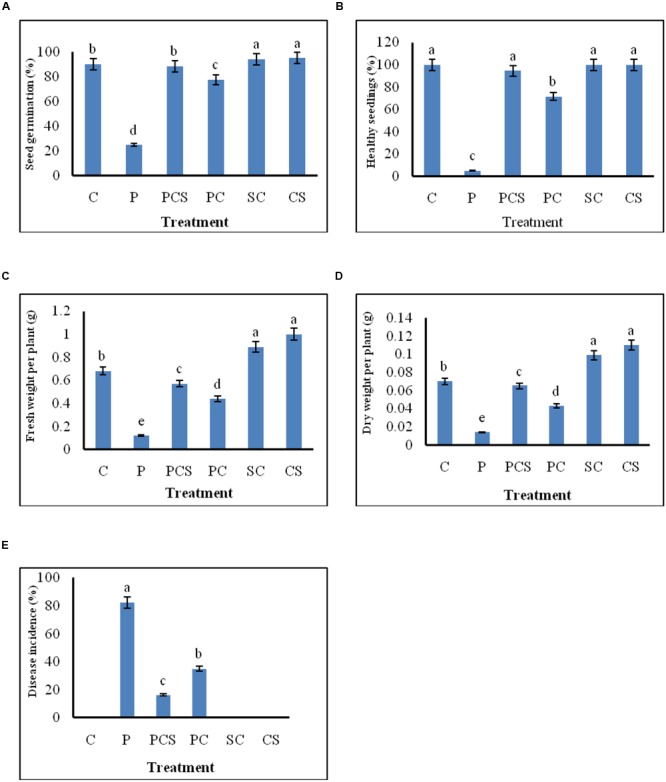
**Effect of *S. hydrogenans* cells and its culture extract as seed dressing on *R. sativus* to control *A. brassicicola* damping off of seedlings. (A)** percentage germination **(B)** emergence of healthy seedlings **(C)** fresh weight per plant **(D)** dry weight per plant **(E)** disease incidence; Data represents means of two experiments with five different treatments of artificially *A. brassicicola* inoculated seeds, each treatment consist of three plates per experiment with 10 seeds per plate, i.e., total 60 seeds for each different treatment. Error bars represent approximate 95% confidence limit. Same letters on the bar are not significantly different according to Tukeys test (*p* ≤ 0.01); C, Control (Water only); P, Pathogen only; PE, Pathogen + Extract; PC, Pathogen + *Streptomyces* cells; SC, *Streptomyces* cells only.

**FIGURE 5:**
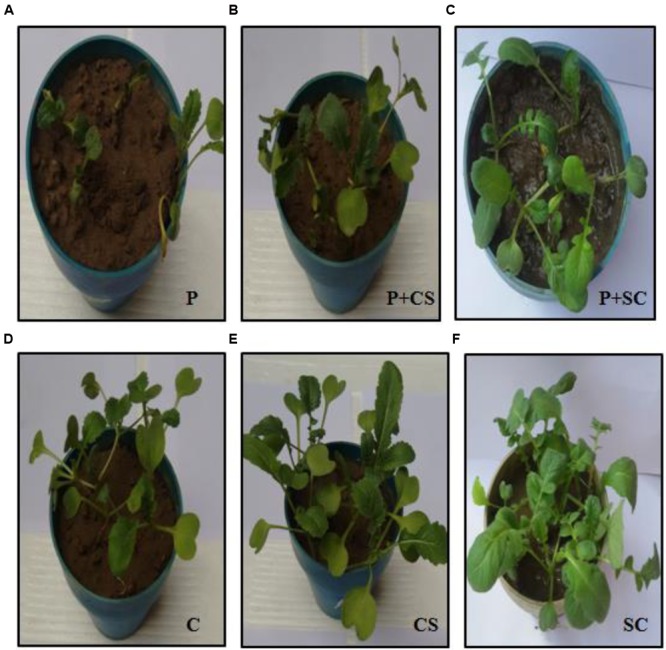
***In vivo* protective effects of *S. hydrogenans* DH16 as seed dressing on radish seeds artificially treated with fungal pathogen (*A. brassicicola*).** Growth of the radish plants (from left to right) after treatment of seeds with **(A)** pathogen **(B)** pathogen and *Streptomyces* cells **(C)** pathogen and extract **(D)** water **(E)** *Streptomyces* extract **(F)** Cells of *Streptomyces*.

The effect of seed treatment on germination of seeds and growth of emerged seedlings was observed for plants grown in soil. Culture supernatant at concentration of 10% (v/v) was selected for further experimentation, since extracellular metabolites at this concentration were found to be strongly inhibitory to the pathogen as shown in *in vitro* blotter test. Significant differences in percentages of seed germination, disease incidence, healthy seedlings, and in fresh and dry weights of emerged plants were found between the different treatments (**Figure 4**). The pathogen infected radish seeds when treated with *Streptomyces* DH16 cells/metabolites, germinated and emerged as healthy seedlings, at significantly (*P* ≤ 0.001) higher rates (70–95%) as compared to seeds treated with pathogen only (5–25%). Similarly, disease severity was also significantly reduced in treated seeds in both the treatments. Although the fresh and dry weights of plants emerged from seeds treated with pathogen and antagonists were lower than the water treated control plants but the plants were higher and stronger than the plants emerged from seeds treated with pathogen only. However, the treatment of seeds with *S. hydrogenans* DH16 cells/culture supernatant only, significantly enhanced the germination rate and growth of emerged plants with significantly (*p* ≤ 0.05) higher fresh and dry weights as compared to control plants (**Figure 5**). When serially diluted aliquots of root segments and rhizosphere soil of plants emerged from streptomycete treated seeds were plated, the cfu counts of 1 × 10^7^/cm and 1 × 10^8^/g soil, respectively, were observed.

### Biocontrol Potential of Culture Supernatant and *Streptomyces* DH16 As Foliar Treatment

**FIGURE 6:**
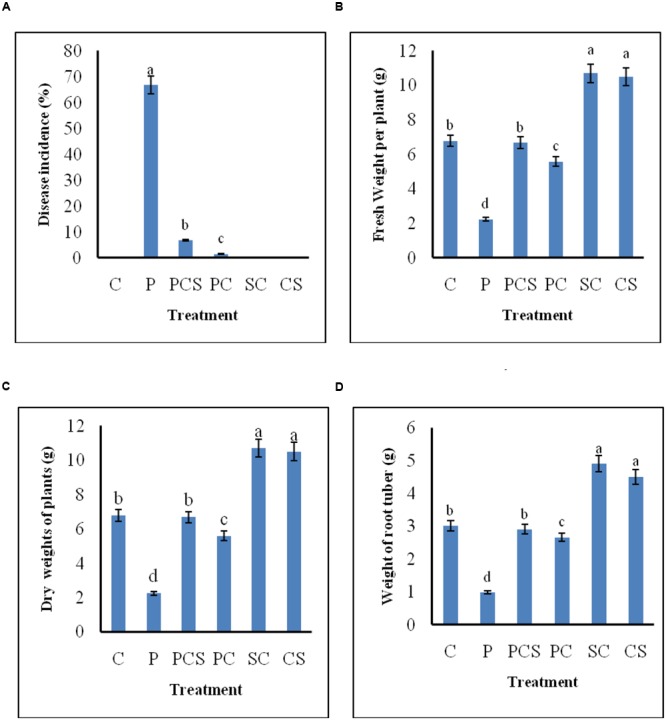
**Effect of *S. hydrogenans* and its culture filtrate as foliar treatments on *R. sativus* to control *A. brassicicola* causing black leaf spot disease. (A)** Percentage of disease incidence **(B)** fresh weight of plants **(C)** dry weight of plants **(D)** weight of root tuber. Error bars represent approximate 95% confidence limit. Same letters on the bar are not significantly different according to Tukeys test (*p* ≤ 0.01); C, Control (Water only); P, Pathogen only; PE, Pathogen + Extract; PC, Pathogen + *Streptomyces* cells; SC, *Streptomyces* cells only.

**FIGURE 7:**
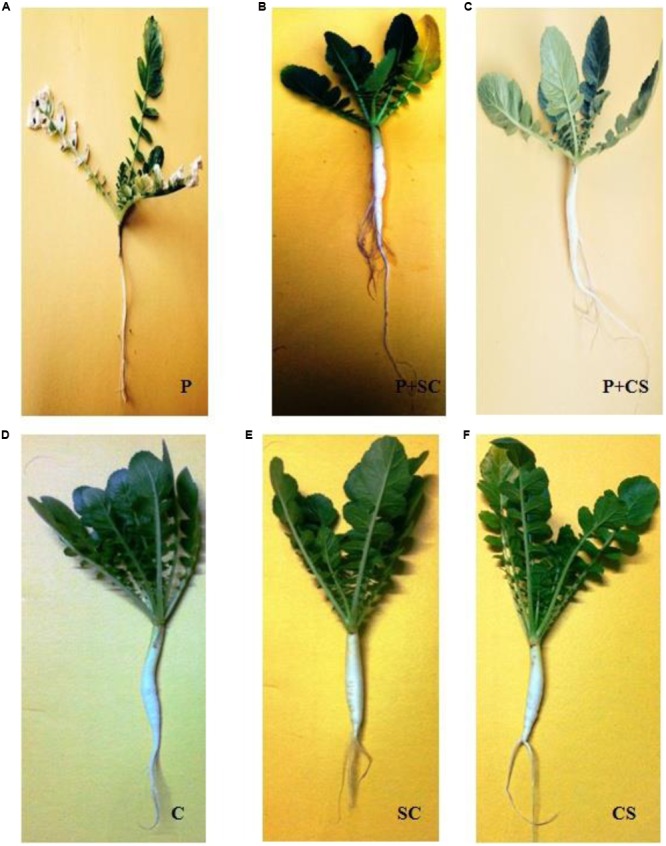
**Biocontrol potential of *S. hydrogenans* DH16 and its culture filtrate as foliar application on *A. brassicicola* causal agent of leaf spot disease in pot experiments.** Radish plants 20 days after inoculation with **(A)** pathogen **(B)** pathogen + *Streptomyces* cells **(C)** pathogen + *Streptomyces* extract **(D)** water only **(E)** *Streptomyces* DH16 only **(F)** *Streptomyces* extract.

**Figure 6** demonstrates the biocontrol ability of *Streptomyces* DH16 and its culture supernatant as effective foliar treatments to control black leaf spot disease on radish leaves. In control plants, the symptoms of disease caused by *A. brassicicola* were clearly visible with 66.81% disease severity and yellowing of leaves and therefore under developed roots. On the other side, symptoms of disease were rarely observed in plants treated with antagonist and its metabolites which significantly reduced the disease severity to 6.78 and 1.47%, respectively (*p* ≤ 0.01). The fresh and dry weights of treated plants were also significantly higher over the pathogen inoculated plants (*p* ≤ 0.05). Weight of tap roots (edible part) in treated plants was comparable to the control plants, in contrast to pathogen treated plants where they were not developed properly. As compared to uninoculated control plants, edible tap roots with high fresh weight were obtained in plants where soil was irrigated with spore suspension/culture supernatant of *S. hydrogenans* DH16 (**Figure 7**).

## Discussion

Biological control of various plant diseases that reduce crop yields has gained the attention of researchers due to its safe and ecofriendly application and therefore, will play vital role in continuous supply of food to the increasing world population. Among various groups of microbial biocontrol agents, actinobacteria especially, number of *Streptomyces* spp. has been reported as potential biological agents to control various phytopathogenic fungi (Mukherjee and Sen, 2006; Prabavathy et al., 2006; Bressan and Figueiredo, 2008; Li et al., 2011).

This study demonstrated *in vitro* and *in vivo* potential of *S. hydrogenans* strain DH16 to control black leaf spot and damping off of *R. sativus* caused by *A. brassicicola*. In the present investigation, *in vitro* studies revealed inhibition of mycelial growth and spore germination of *A. brassicicola* in the presence of culture supernatant of *S. hydrogenans.* With increasing concentration of culture supernatant, suppression of both mycelial growth and conidial germination increased and almost complete inhibition was observed at concentration of 20%. These results coincides with earlier reports of Prapagdee et al. (2008) and Li et al. (2011) who also studied the effect of culture filtrates of *Streptomyces* spp., *S. hygroscopicus*, and *S. globisporus* JK-1, on the mycelial growth of *Colletotrichum gloeosporioides* and *Sclerotium rolfsii*, and *Magnaporthe oryzae*, respectively. In contrast, fistupyrone, a metabolite from endophytic *Streptomyces* sp. TP-A0569, showed inhibitory effects on spores of *A. brassicicola* but could not inhibit its mycelial growth on PDB and PDA even at concentration of 1000 ppm (Aremu et al., 2003).

Antibiotics from actinobacteria antagonize phytopathogenic fungi by inducing various morphological alterations such as stunting, distortion, swelling, hyphal protuberances in mycelial structure or the highly branched appearance of fungal germ tubes (Gunji et al., 1983). Getha and Vikineswary (2002) observed severe morphological changes such as swellings, distortions, and excessive branching in *Fusarium oxysporum* f.sp. *cubense* race 4 caused by extracellular metabolites from *S. violaceusniger* strain G10. Prapagdee et al. (2008) also reported the absence of *C. gloeosporioides* conidia as one of the malformations caused by *S. hygroscopicus* and its sterile culture filtrates.

Similarly, in the present study microscopic observations of fungal mycelia from the margins of the inhibition zones (resulted from culture broth of strain DH16) revealed severe structural alterations in vegetative cells and spores, which indicated that metabolites probably attack the cell wall/cell membrane. These findings further confirmed the antibiosis as the selective mechanism of antifungal activity of strain DH16. One of the antifungal compounds of *S. hydrogenans* strain DH16 has been purified and characterized to be 10-(2,2-dimethyl-cyclohexyl)-6,9-dihydroxy-4,9-dimethyl dec-2-enoic acid methyl ester (SH2; Kaur et al., 2016b).

The mechanism of antibiosis is considered to be advantageous in biological control of plant diseases because antimicrobials can diffuse rapidly in nature, and thus, direct contact between the pathogen and antagonist is not indispensable (Hajlaou et al., 1994). In addition, loss of pigmentation (melanin) in hyphae as well as spores of *A. brassicicola* was also observed. Loss in integrity of cell wall/cell membrane was further confirmed by leakage of cellular materials (electrolytes) indicated by changes in extracellular conductivity.

Organic farming needs alternative seed treatments to eliminate or at least effectively reduce the seed-borne pathogens (Amein et al., 2011). Biological seed treatments with microbial antagonists are attractive alternatives to the chemical pesticides because the latter lead to changes in the metabolic profiles of rhizosphere biodiversity (Correa et al., 2009) whereas the bacterization of seeds with microorganisms does not alter the beneficial rhizosphere bacterial community. Moreover, the seed bacterization method has been proved to be effective as the biocontrol agent can rapidly and extensively grow and cover the surface of the seeds and can protect the plants from invading soil-borne pathogens (Kanini et al., 2013).

In recent years, the use of crude secondary metabolites produced by *Streptomyces* spp. are also gaining importance in crop protection and these metabolites may be a supplement or an alternative to chemical pesticides (Zacky and Ting, 2013; Harikrishnan et al., 2014). Therefore, in this study both the cells as well as extracellular antifungal metabolites of *S. hydrogenans* strain DH16 were evaluated for their *in vivo* biocontrol potential against *A. brassicicola.* The treatment of pathogen infested radish seeds with streptomycete cells/culture supernatant lead to statistically significant (*p* ≤ 0.05) improvement in seed germination, seedling vigor and plant weight, in both blotter assay as well as in *in vivo* pot experiments and thus reduced the frequency rate of damping off of seedlings.

In addition to disease control, cells as well as culture supernatant of *Streptomyces* DH16 significantly enhanced vigor index and other agronomic parameters (fresh and dry weights) over uninoculated control when applied as seed dressing. These results indicate the plant growth promoting prospect of *S. hydrogenans* DH16 in the absence of pathogen stress which is to its root colonizing ability as indicated by the adherence of bacterium to the roots and associated soil. The ability to colonize roots is an important trait of plant growth promoting microorganisms for their beneficiary effects (Bouizgarne, 2013) and is also related to the effectiveness of biocontrol activity against pathogens (Bull et al., 1991). To our best knowledge, only one report is available in literature where seed treatment is used to control *A. brassicicola.* In 1987, Tahvonen and Avikainen reported the biocontrol of seed borne *A. brassicicola* of cruciferous plants with a powdery preparation of a *Streptomyces* sp. However, till date no biological seed treatment method has been used commercially.

Furthermore, it is challenging to obtain pathogen free seeds of *Brassica* spp., due to the lack of foliar fungicides (Kohl et al., 2010). *Alternaria* species have also been reported to develop resistance against dicarboximides and phenylpyrroles (Iacomi-Vasilescu et al., 2004). Therefore, cells and metabolites of *S. hydrogenans* strain DH16 were also evaluated as foliar application to control black leaf spot by *A. brassicicola*. Both the cells as well as metabolites caused reduction of black leaf spot on *R. sativus* and significantly increased the size and weight of swollen stem as campared to negative control (fungal inoculated plant). In addition, plants irrigated with spore suspension/culture supernatant of streptomycete showed growth enhancement over the untreated control plants which further confirm the PGP potential of the *S. hydrogenans* DH16. Fistupyrone is the only metabolite reported earlier from *Streptomyces* sp. which displayed *in vivo* suppression of black leaf spot caused by *A. brassicicola* on the seedlings of Chinese cabbage (Igarashi et al., 2000).

The outcomes of the current study clearly indicate the great potential of *S. hydrogenans* DH16 (a less studied streptomycete) as another potent biocontrol agent which can be used to control both seed borne as well as foliar pathogen, *A. brassicicola*. The importance of the study lies in the fact that both spores/mycelium and extracellular metabolites of this streptomycete reduced the incidence of *A. brassicicola*, therefore, this strain might be used as a biofungicide in two forms, one having spores and other containing antifungal metabolites. Additionally, this strain is also having insecticidal (Kaur et al., 2014), nematicidal (Kaur et al., 2016a) and plant growth promoting activities (data communicated) which make it superior over already reported chemical and biofungicides (mycostop, fistupyrone, actinovate, and rhizovit) and thus can also be used as bioinsecticide and bio-fertilizer in addition to biofungicide.

## Author Contributions

TK was involved in the planning and execution of the research work; analysis and interpretation of the data; manuscript writing following the suggestions of the research supervisor. RM as research supervisor of TK was involved in the design and planning of research work; analysis and interpretation of data; drafting as well as critical editing of the manuscript for intellectual subject matter. Both the authors approved the final version of the manuscript for publication and agreed to be accountable for all aspects of the work in ensuring that questions related to the accuracy or integrity of any part of the work are appropriately investigated and resolved.

## Conflict of Interest Statement

The authors declare that the research was conducted in the absence of any commercial or financial relationships that could be construed as a potential conflict of interest.
